# Sickle cell disease and opioid overdose outcomes in the United States: a nationwide analysis

**DOI:** 10.1007/s00277-025-06236-x

**Published:** 2025-03-10

**Authors:** Anna L. Bode, Oscar F. Borja-Montes, Mohammed A. Quazi, Aqsa Mumtaz, Amir H. Sohail, Christopher R. Smith, Muhammad Rizwan Khawaja, Abu Baker Sheikh

**Affiliations:** 1 Department of Internal Medicine, University of New Mexico, Albuquerque, NM USA; 2 Department of Family and Community Health, West Virginia University, Morgantown, WV USA; 3 Montefiore St. Luke’s Cornwall Hospital, Newburg, NY USA; 4 Department of Surgery, University of New Mexico, Albuquerque, NM USA; 5 Associate Clinical Investigator, Honor Health Research Institute, Scottsdale, AZ USA

**Keywords:** Sickle cell disease, Opioid overdose, Mortality, In-hospital complications, Venous thromboembolism

## Abstract

Sickle cell disease (SCD) predominantly affects individuals of African descent and is characterized by frequent painful vaso-occlusive crises, often requiring opioid management. With the opioid epidemic raising concerns about opioid overdose, this study examines in-hospital outcomes among SCD and non-SCD patients hospitalized for opioid overdose. Using the National Inpatient Sample (NIS) from 2016 to 2021, we analyzed 479,175 opioid overdose hospitalizations, including 1,315 (0.3%) with a concomitant diagnosis of SCD. Propensity score matching was used to balance demographics, comorbidities, and hospital characteristics. SCD patients were younger (45.3% aged 30–49 vs. 33.1%, *p* < 0.001), predominantly of African descent (92.1% vs. 14.1%, *p* < 0.001), and more often from lower-income households (58.4% vs. 35.2%, *p* < 0.001). SCD patients had lower rates of mechanical ventilation (aOR: 0.7, 95% CI: 0.6–0.9) and anoxic brain injury (aOR: 0.5, 95% CI: 0.4–0.8) but experienced longer hospital stays and higher costs. No significant differences in in-hospital mortality were observed (aOR: 0.89, 95% CI: 0.7–1.1, *p* = 0.34). These findings emphasize the need for a nuanced approach to managing SCD patients during opioid overdose hospitalizations, focusing on mitigating complications, addressing prolonged hospital stays and higher costs, and reducing healthcare disparities through tailored strategies informed by the unique needs of this population.

**Supplementary Information:**

The online version contains supplementary material available at 10.1007/s00277-025-06236-x.

## Introduction

Sickle cell disease (SCD) is an inherited hematologic disorder characterized by a missense mutation in the human beta-globin gene, leading to the formation of the sickle hemoglobin variant HbS [1]. Primarily affecting individuals of African descent, SCD is the most common hemoglobinopathy, with millions of patients impacted globally and approximately 300,000 new cases emerging every year [2, 3]. In the United States, epidemiological studies estimate that at least 100,000 individuals live with SCD [4]. The disease imposes a significant economic burden on healthcare systems, as patients frequently experience catastrophic pain events due to vaso-occlusive crises (VOC) [3, 5]. These crises can be debilitating and often necessitate aggressive pain management.

Opioid medications are commonly employed to manage severe pain in SCD patients, yet there is increasing concern about the risk of opioid use disorder and the potential for overdose within this population. The opioid overdose crisis is a well-recognized public health issue that has been exacerbated since the onset of the opioid epidemic. In 2017, the cost of opioid use disorder and fatal opioid overdose in the United States was estimated to be USD 1.02 trillion [6, 7]. This epidemic has created barriers to adequate pain control for chronic conditions such as SCD, leading to evidence of pain undertreatment even before the opioid crisis began in the late 1990s [8, 9]. Several factors contribute to the undertreatment of pain in SCD patients, including knowledge deficits among healthcare providers, the influence of governmental regulations, fears of addiction, respiratory depression, and opioid misuse [9, 10]. Additionally, patients with SCD are more likely to reside in underserved areas, facing further barriers to healthcare access. For instance, a study in New York City revealed that pharmacies in predominantly nonwhite neighborhoods often do not stock sufficient medications to treat severe pain adequately [11].

Patients with SCD are also vulnerable to life-threatening complications such as acute chest syndrome [12, 13]. This condition raises concerns among physicians regarding opioid prescription due to the risk of exacerbating respiratory depression in hypoxic patients [14]. A recent observational study examining mortality trends over the past 40 years in SCD found a reduced burden of drug-related complications in SCD-related deaths compared to similar non-SCD-related deaths, despite the routine use of opioids to manage acute and chronic SCD-related pain [15]. Nonetheless, ongoing concerns about opioid-related complications in SCD patients persist when addressing acute VOC.

Building upon these concerns, our study aims to assess outcomes in patients hospitalized with opioid overdose, both with and without SCD, using data from the National Inpatient Sample (NIS). The primary outcome of interest is in-hospital mortality, with secondary outcomes including a range of in-hospital complications (such as mechanical ventilation, acute liver failure, sudden cardiac arrest, acute kidney injury, and venous thromboembolism) and health services utilization (i.e. cost of hospitalization and length of stay). By examining these outcomes, we aim to provide a comprehensive understanding of the impact of opioid overdose on SCD patients compared to their non-SCD counterparts.

## Materials and methods

### Data

This study utilized the NIS dataset from 2016 to 2021, the largest publicly accessible all-payer inpatient database in the United States. The NIS captures data from approximately 20% of all hospital discharges nationwide and is weighted to represent the entire U.S. inpatient population. Survey weights provided by the NIS were incorporated into all statistical modeling and testing. Additional information on the NIS design can be found at https://www.hcup-us.ahrq.gov/ [16, 17]. Since the data are deidentified, this study was exempt from institutional review board approval.

### Study population

We identified hospitalized patients with a primary diagnosis of opioid overdose using International Classification of Diseases, Tenth Revision (ICD-10) codes (refer to Supplemental Table 1). Patients with sickle cell disease (SCD) were identified using ICD-10 codes and patients with sickle cell trait (HbAS) were excluded. We also excluded patients under 16 years of age, those with missing data for key variables, and patients with a concurrent COVID-19 diagnosis.

### Covariates

The NIS dataset contains information on in-hospital outcomes, procedures, and other discharge-related details. Covariates were categorized into three groups:

1. **Patient-level variables**: Age, race, sex, comorbidities, insurance status, median income in the patient’s ZIP code, and disposition.
2. **Hospital-level variables**: Location, teaching status, bed size, and region.
3. **Illness severity variables**: Length of stay (LOS), mortality, hospitalization cost, Elixhauser comorbidity score, vasopressor use, mechanical ventilation (invasive and non-invasive), acute kidney injury (AKI), venous thromboembolism (VTE), sudden cardiac arrest, cardiac dysrhythmias, AKI requiring hemodialysis, acute liver failure, and anoxic brain injury.

### Outcomes

The primary outcome was in-hospital mortality. Secondary outcomes included in-hospital complications and healthcare resource utilization metrics, such as length of stay and inflation-adjusted hospitalization charges. Hospitalization charges were adjusted to 2021 dollars using the Consumer Price Index.

### Statistical analysis

In Tables 1 and 2, we report the *p*-values from chi-squared tests for independence between categorical variables in the two survey-weighted cohorts (opioid overdose with SCD and opioid overdose without SCD). Multivariable logistic regression models, incorporating survey weights, were developed to examine complications, as shown in Table 2. These models report adjusted odds ratios (aOR) for the two cohorts, along with 95% confidence intervals (CIs) and *p*-values. Patient-related, hospital-related, and Elixhauser comorbidities were used as predictor variables to adjust the odds ratios in the logistic regression models for each complication. Stepwise regression with a threshold of *p*-value < 0.05 was applied to retain predictors in the final multivariable weighted logistic regression models.

**Table 1:** Opioid overdose patients with sickle cell disease (SCD+) and without (w/o) SCD: patient-level characteristics

Characteristics	Opioid Overdose and SCD -	Opioid Overdose and SCD +	*p* value
*n* = 479,175	*n* = 477,860 (99.73%)	*n* = 1,315 (0.27%)	
**Gender (%)**			< 0.001
Female	229,460 (48.02%)	715 (54.37%)	
Male	248,400 (51.98%)	600 (45.63%)	
**Mean Age Years (SD)**			
Female	51.87 (17.14)	42.35 (14.29)	
Male	45.78 (16.78)	39.39 (13.42)	
**Age Groups (%)**			< 0.001
16–29	81,900 (17.14%)	305 (23.19%)	
30–49	154,725 (32.38%)	640 (48.67%)	
50–69	185,445 (38.81%)	335 (25.48%)	
>= 70	55,790 (11.67%)	35 (2.66%)	
**Race (%)**			< 0.001
Asian or Pacific Islander	3,600 (0.75%)	5 (0.38%)	
Black	62,695 (13.12%)	1,185 (90.11%)	
Hispanic	38,325 (8.02%)	60 (4.56%)	
Native American	3,810 (0.8%)	X*	
Other	12,020 (2.52%)	35 (2.66%)	
White	357,410 (74.79%)	30 (2.28%)	
**Median Household Income**			< 0.001
<= 51,999	165,420 (34.62%)	745 (56.65%)	
52 K – 65,999	126,890 (26.55%)	275 (20.91%)	
66 K – 87,999	108,050 (22.61%)	185 (14.07%)	
>= 88 K	77,500 (16.22%)	110 (8.37%)	
**Insurance Status (%)**			< 0.001
Medicaid	159,305 (33.34%)	470 (35.74%)	
Medicare	162,975 (34.11%)	640 (48.67%)	
Other	14,040 (2.94%)	20 (1.52%)	
Private Insurance	86,610 (18.12%)	145 (11.03%)	
Self-pay	50,480 (10.56%)	40 (3.04%)	
**Hospital Division (%)**			< 0.001
East North Central	76,640 (16.04%)	205 (15.59%)	
East South Central	35,675 (7.47%)	80 (6.08%)	
Middle Atlantic	66,070 (13.83%)	210 (15.97%)	
Mountain	31,190 (6.53%)	45 (3.42%)	
New England	27,745 (5.81%)	25 (1.9%)	
Pacific	53,210 (11.14%)	100 (7.6%)	
South Atlantic	117,385 (24.56%)	445 (33.84%)	
West North Central	27,890 (8.8%)	85 (6.46%)	
West South Central	42,055 (10.73%)	120 (9.13%)	
**Hospital Bedsize (%)**			< 0.001
Large	227,145 (47.53%)	725 (55.13%)	
Medium	145,525 (30.45%)	360 (27.38%)	
Small	105,190 (22.01%)	230 (17.49%)	
**Hospital Teaching Status (%)**			< 0.001
Rural	38,535 (8.06%)	35 (2.66%)	
Urban nonteaching	106,550 (22.3%)	145 (11.03%)	
Urban teaching	332,775 (69.64%)	1,135 (86.31%)	
**Comorbidities (%)**			
HTN	213,710 (44.72%)	555 (42.21%)	0.067
Diabetes (2)	92,160 (19.29%)	185 (14.07%)	< 0.001
Cancer (5)	19,415 (4.06%)	35 (2.66%)	0.010
Obesity	66,925 (14.01%)	180 (13.69%)	0.741
Cannabis Use	53,280 (11.15%)	120 (9.13%)	0.020
Smoking	250,895 (52.5%)	550 (41.83%)	< 0.001
Alcohol	70,640 (14.78%)	35 (2.66%)	< 0.001
Chronic Pulmonary Disease	129,020 (27%)	340 (25.86%)	0.351
Hypothyroidism	45,495 (9.52%)	70 (5.32%)	< 0.001
Autoimmune	15,435 (3.23%)	50 (3.8%)	0.241
Depression	145,895 (30.53%)	300 (22.81%)	< 0.001
AIDS	6,155 (1.29%)	25 (1.9%)	0.049
Dementia	13,600 (2.85%)	25 (1.9%)	0.040
Homeless	16,530 (3.46%)	20 (1.52%)	< 0.001

*AIDS: Acute immunodeficiency syndrome; HTN: Hypertension; SCD: Sickle cell disease. * X: Too small to report per NIS data

**Table 2:** In-hospital outcomes in opioid overdose patients with sickle cell disease(SCD+) and without (w/o) SCD

	Opioid Overdose and SCD -	Opioid Overdose and SCD +	Odds Ratio for SCD+	95% CI Lower Limit	95% CI Upper Limit	*P* value
**In hospital Mortality (%)**	21,500 (4.5%)	45 (3.42%)	0.85	0.43	1.67	0.639
**Complications (%)**						
Sudden Cardiac Arrest	22,695 (4.75%)	65 (4.95%)	1.04	0.59	1.83	0.901
Vasopressor use	9,515 (1.99%)	15 (1.14%)	0.49	0.16	1.54	0.221
AKI	157,220 (32.9%)	425 (32.32%)	0.91	0.70	1.18	0.468
Invasive Mechanical Ventilation	109,040 (22.82%)	135 (10.27%)	0.38	0.26	0.57	0.000
Non-Invasive Mechanical Ventilation	27,130 (5.68%)	65 (4.94%)	0.88	0.50	1.55	0.659
Hemodialysis	13,675 (2.86%)	95 (7.22%)	1.35	0.83	2.19	0.227
VTE	8,970 (1.88%)	60 (4.56%)	2.30	1.28	4.12	0.005
Anoxic brain Damage	22,885 (4.79%)	20 (1.52%)	0.28	0.10	0.74	0.011
Cardiac Dysrhythmia	47,285 (9.9%)	105 (7.98)	1.38	0.87	2.19	0.168
Acute Liver Failure	16,555 (3.46%)	40 (3.04%)	0.94	0.46	1.92	0.870

Mean Inflation-Adjusted Cost ($)	$53,486.23	$76,602.76	< 0.001			
	Adjusted Inflation-adjusted cost*= $5,381.43 higher for SCD+					
**Mean Length of stay (days)**	4.60	7.16	< 0.001			
	Adjusted length of stay*= 2.76 days higher for SCD+					

Disposition			
Characteristics	Opioid Overdose and SCD -	Opioid Overdose and SCD +	*p* value
*n* = 479,175	*n* = 477,860 (99.73%)	*n* = 1,315 (0.27%)	
**Disposition (%)**			< 0.001
Against Medical Advice	47,430 (9.93%)	55 (4.18%)	
Died in Hospital	21,500 (4.5%)	45 (3.42%)	
Home health care	44,560 (9.32%)	135 (10.27%)	
Routine	261,240 (54.67%)	925 (70.34%)	
Transfer other	92,095 (19.27%)	115 (8.75%)	
Transfer to short-term hospital	10,995 (2.3%)	40 (3.04%)	

* Adjusted for Age, Hospital bed-size, Race, Gender, Hospital location, Hospital teaching status, Hospital region, Median household income, Expected primary payer (insurance status), Elixhauser comorbidities. * AKI: Acute kidney injury; PSM: Propensity score matching; SCD: Sickle cell disease; VTE: Venous thromboembolism;

For inflation-adjusted hospital charges and length of stay, we used a single weighted multivariate linear regression model with the same predictor variables as in the logistic regression models, applying the same *p*-value threshold for predictor retention. The multivariate linear regression model reports the mean differences in length of stay and mean inflation-adjusted total hospitalization charges between the two cohorts (Table 2).

Additionally, a propensity-matched sample was extracted from the two cohorts using the predictor variables from the statistical models. Chi-squared tests and regression analyses were repeated on the propensity-matched cohorts to validate the findings. Statistical analyses were conducted using R Programming Language (version 4.4.1) and Python (version 3.11), while data curation was performed using SAS (version 9.2).

## Results

### Demographics and baseline comorbidities

Our study population included a total of 479,175 hospitalized patients with opioid overdose, out of which 1,315 (0.27%) had a concomitant history of sickle cell disease (SCD). Patients with SCD and opioid overdose were more frequently female (54.37% vs. 48.02%, *p* < 0.001). The prevalence of opioid overdose and SCD was significantly higher among patients aged 30–49 years (48.67% vs. 32.38%, *p* < 0.001) and 16–29 years (23.19% vs. 17.14%, *p* < 0.001), with mean ages of 42.35 ± 14.3 years for females and 39.39 ± 13.4 years for males. This cohort was predominantly of African descent (90.11% vs. 13.07%, *p* < 0.001) and more likely to have a median household income below USD 50,000 (56.65% vs. 34.6%, *p* < 0.001) compared to patients with opioid overdose without SCD.

Patients with SCD and opioid overdose were less likely to smoke (41.83% vs. 52.5%, *p* < 0.001), consume alcohol (2.66% vs. 14.78%, *p* < 0.001), or have a history of cannabis consumption (9.13% vs. 11.15%, *p* = 0.020). They were also less likely to have depression (22.81% vs. 30.53%, *p* < 0.01), dementia (1.90% vs. 2.85%, *p* = 0.040), cancer (3.66% vs. 4.06%, *p* = 0.010), hypothyroidism (5.32% vs. 9.53%, *p* < 0.01), and homelessness (1.52% vs. 3.46%, *p* < 0.001).

Geographically, most patients in both groups were treated at urban teaching hospitals (86.31% vs. 69.62%). A larger proportion of patients with SCD were recipients of Medicare (48.67% vs. 34.11%, *p* < 0.001). The baseline demographics of the study cohorts are summarized in Table 1, with the initial baseline characteristics of the matched cohort post-propensity matching detailed in Supplementary Table S2.

### In-hospital mortality

A multivariable weighted logistic regression analysis, adjusted for age, hospital bed capacity, race, gender, hospital location, teaching status, regional placement, median household income, anticipated primary payer, and Elixhauser comorbidities (Table 2), revealed an in-hospital mortality rate of 3.42% for patients with SCD compared to 4.5% for non-SCD patients with opioid overdose (aOR: 0.85, 95% CI: 0.43–167; *p* = 0.639. This difference, however, did not show statistical significance.

Propensity score matching (PSM) was performed considering variables such as patient’s age, gender, race, income, insurance status, and Elixhauser comorbidities (refer to Supplementary Table S2). After the PSM process, each group (those with SCD and those without) comprised 2,630 patients (Supplementary Table S2). The total number of patients with in-hospital mortality was 75, with 30 (2.8%) from the non-SCD group and 45 3.42%) from the SCD group. The aOR was calculated to be 1.28 (95% CI 0.42–3.96) with a *p*-value of 0.664, indicating that the difference in mortality between the two groups was not statistically significant (Table 3).

**Table 3:** Propensity 1:1 matched in hospital outcomes in opioid overdose patients with sickle cell disease(SCD+) and without (w/o) SCD

PSM	Opioid Overdose and SCD -	Opioid Overdose and SCD +	Odds Ratio for SCD+	95% CI Lower Limit	95% CI Upper Limit	*P* value
**In Hospital Mortality**	30 (2.28%)	45 (3.42%)	1.28	0.42	3.96	0.664
**Complications (%)**						
Sudden Cardiac Arrest	65 (4.94%)	65 (4.94%)	0.98	0.44	2.17	0.961
Vasopressor use	40 (3.04%)	15 (1.14%)	0.33	0.08	1.29	0.110
AKI	455 (34.6%)	425 (32.32%)	0.89	0.61	1.30	0.536
Invasive Mechanical Ventilation	310 (23.57%)	135 (10.27%)	0.34	0.21	0.56	< 0.001
Non-Invasive Mechanical Ventilation	55 (4.18%)	65 (4.94%)	1.25	0.51	3.09	0.622
Hemodialysis	65 (4.94%)	95 (7.22%)	1.60	0.74	3.45	0.234
VTE	55 (4.18%)	60 (4.56%)	1.11	0.48	2.56	0.810
Anoxic brain Damage	75 (5.7%)	20 (1.52%)	0.16	0.05	0.56	0.004
Cardiac Dysrhythmia	100 (7.6%)	105 (7.98%)	1.09	0.56	2.12	0.800
Acute Liver Failure	60 (4.56%)	40 (3.04%)	0.66	0.26	1.64	0.371

Mean Inflation-Adjusted Cost ($)	$57,649.83	$76,602.76	0.009			
	Adjusted Inflation-adjusted cost*= $4,333.40 higher for SCD+					
Mean Length of stay (days)	4.80	7.16	< 0.001			
	Adjusted length of stay*= 2.32 days higher for SCD+					

PSM Disposition			
**Characteristics**	**Opioid Overdose and SCD -**	**Opioid Overdose and SCD +**	**p value**
*n* = 3,**320**	*n* = 1,**315 (50.0%)**	*n* = 1,**315 (50.0%)**	
**Disposition (%)**			0.272
Against Medical Advice	30 (2.28%)	55 (4.18%)	
Died in Hospital	30 (2.28%)	45 (3.42%)	
Home health care	130 (9.89%)	135 (10.27%)	
Routine	880 (66.92%)	925 (70.34%)	
Transfer other	180 (13.69%)	115 (8.75%)	
Transfer to short-term hospital	65 (4.94%)	40 (3.04%)	

* Adjusted for Age, Hospital bed-size, Race, Gender, Hospital location, Hospital teaching status, Hospital region, Median household income, Expected primary payer (insurance status), Elixhauser comorbidities. * AKI: Acute kidney injury; PSM: Propensity score matching; SCD: Sickle cell disease; VTE: Venous thromboembolism

### In-hospital complications

The incidence of opioid overdose related-complications such as invasive mechanical ventilation (22.82% vs. 10.27%, aOR: 0.38 [95% CI 0.26–0.567, *p*-value < 0.001) and anoxic brain damage (4.79% vs. 1.52%, aOR: 0.28, [95% CI 0.10–0.71, *p*-value: 0.011) were significantly higher in the group without SCD compared to the group with SCD, whereas in the SCD group venous thromboembolism was higher (4.56% vs. 1.88%, aOR: 2.30, [95% CI 1.28–4.12], *p*-value: 0.005).

The study also analyzed the incidence of other complications including sudden cardiac arrest (4.75% vs.4.92%, aOR: 1.04, [95% CI 0.59–1.83], *p*-value: 0.901), vasopressor use (1.99% vs. 1.14%, aOR: 0.49, [95% CI 0.16–1.54], *p*-value: 0.221), acute kidney injury (32.90% vs. 32.32%, aOR: 0.91, [95% CI 0.70–1.18], *p*-value: 0.468), non-invasive mechanical ventilation (5.68% vs. 4.94%, aOR: 0.88, [95% CI 0.50–1.55], *p*-value: 0.659), cardiac dysrhythmia (9.90% vs. 7.98%, aOR: 1.38, [95% CI 0.87–2.19], *p*-value: 0.168) and acute liver failure (3.46% vs. 3.04%, aOR: 0.94, [95% CI 0.46–1.92], *p*-value: 0.870). However, there were no statistically significant differences in the incidence of these complications between the two groups of patients (Table 2).

After PSM, patients with opioid overdose without SCD group continued to have a greater incidence of invasive mechanical ventilation (23.57% vs. 10.27%, aOR: 0.34, [95% CI 0.21–0.56, *p*-value < 0.001) and anoxic brain damage (5.7 vs. 1.52%, aOR: 0.16, [95% CI 0.05–0.56], *p*-value: 0.04, yet, no marked differences identified in sudden cardiac arrest (4.94% vs. 4.93%, aOR: 0.98, [95% CI 0.44–2.17], *p*-value: 0.961), acute kidney injury (32.32% vs. 34.34%, aOR: 0.89, [95% CI 0.61–1.30], *p*-value: 0.536), VTE (4.56% vs. 4.18%, aOR: 1.11 [95% CI 0.48–2.56], *p*-value: 0.810) and other complications (Table 3).

### In-hospital quality measures and disposition

Patients with opioid overdose and SCD had a statistically significant increased mean length of stay (7.16 days vs. 4.60 days, adjusted length of stay 2.76 days higher for those with SCD, *p* < 0.001) compared to non-SCD patients with opioid overdose. This cohort also had a statistically significant difference in total hospitalization charge (USD 76,602.76 vs. USD 53,486.23, adjusted inflation-adjusted cost of USD 5,381.43 higher for SCD patients, *p* < 0.001).

Additionally, patients with SCD were less likely to leave against medical advice (4.18% vs. 9.93%, *p* < 0.001) and had higher percentages of discharge to routine care (70.34% vs. 54.67%, *p* < 0.001) and higher rates of home health care services (10.27% vs. 9.32%, *p* < 0.001).

Following PSM, these differences remained evident. Patients with opioid overdose and SCD continued to have an increased length of stay by 2.32 days (7.16 days vs. 4.80 days, adjusted *p* < 0.001) and a higher hospitalization charge by USD 4,333.40 (USD 76,602.76 vs. USD 57,649.83, *p* = 0.09), statistically significant. Their discharge patterns were consistent: lower rates of discharge against medical advice (2.28% vs. 4.18`%, *p* < 0.001) and higher rates of discharge to routine care (70.34% vs. 66.92%, *p* < 0.001) (Table 3). Both differences were statistically significant.

## Discussion

Vaso-occlusive crises are a leading cause of emergency department visits and hospitalizations among sickle cell disease patients. Despite existing guidelines, pain control in SCD remains suboptimal. In our nationwide analysis, we observed no significant difference in in-hospital mortality between patients with SCD and those without SCD who were admitted for opioid overdose. Additionally, while the incidence of invasive mechanical ventilation and anoxic brain injury was significantly lower in the SCD group, other complications, such as venous thromboembolism, acute kidney injury, and sudden cardiac arrest, did not show statistically significant differences between the two groups.

Effective management of VOCs relies on liberal fluid administration and adequate pain management [18, 19]. Prompt recognition and early intervention with analgesics can alleviate pain and reduce the length of hospital stays. However, individuals with SCD often face significant stigma, being labeled as “frequent fliers,” “drug seekers,” or “difficult patients” [20–24]. The opioid epidemic then worsened suspicions and accusations toward patients seeking opioid prescriptions, exacerbating their challenges. A 2024 study by Kang et al. found that the 2016 CDC opioid prescribing guidelines led to a significant decrease in opioid dispensation dosage and days per prescription for SCD patients, alongside a significant increase in hospitalizations for VOCs [25]. While the association between pain and SCD is well-recognized, accessing appropriate pain medications, particularly opioids, remains a formidable barrier for SCD patients [25]. Despite the potential for adverse effects, opioids are effective in alleviating SCD-related pain, sometimes necessitating higher-than-normal dosages, which can increase the risk of overdose and mortality. However, our study reveals no significant difference in in-hospital mortality between SCD and non-SCD patients admitted for opioid overdoses. This suggests that while opioid overdose can induce respiratory depression leading to death, outcomes between SCD and non-SCD patients show no notable difference.

Similarly, Ruta et al. analyzed CDC data, finding that from 1999 to 2013, out of 174,959 opioid overdose deaths, only 95 were SCD patients [26]. They also found that opioid-related death rates were lower in SCD patients compared to those with other medical conditions such as migraine, fibromyalgia, and low back pain. Ballas et al. concluded that while opioids are used at higher doses in SCD patients, they are not the leading cause of death in this population, with circulatory issues, infections, respiratory complications, genitourinary problems, and VOCs/acute chest syndrome ranking higher [27]. This is consistent with the 2020 Payne et al. study, which also reported that current SCD deaths are more likely to be related to disease complications rather than drug-related complications [15].

Our study also indicates that non-SCD patients exhibit higher rates of invasive ventilation and anoxic brain injury compared to SCD patients. This discrepancy can be attributed to the heightened opioid tolerance observed in SCD patients due to increased drug clearance, as illustrated in pharmacokinetic studies [28, 29]. In contrast, non-SCD patients may experience severe respiratory depression and hypoxia-associated complications despite receiving comparable opioid dosages. Building on this research, Khan et al. investigated whether patients with SCD who require higher opioid doses to achieve comparable analgesia necessitate more naloxone than the general population [30]. They reported relatively low naloxone usage among SCD patients despite their generally higher opioid usage. These studies highlight how differences in pharmacokinetics and drug clearance can potentially reduce the risk of opioid overdose in SCD patients. Furthermore, our database could not distinguish between prescription and illicit opioid sources, but SCD overdoses are likely more often from prescription opioids, which could also account for differences in complication rates. Regarding VTE, our study focused on opioid overdose hospitalizations and examined VTE as an inpatient complication within this specific context. While VTE is a well-established risk in patients with SCD due to factors like hypercoagulability, antiphospholipid antibodies, low levels of protein C and S, and post-splenectomy thrombocytosis [31, 32], our findings showed no significant difference in the incidence of VTE between SCD and non-SCD patients after PSM. This observation suggests that the acute context of opioid overdose hospitalizations may influence the typical risk profile for VTE in SCD patients, potentially due to differences in admission circumstances, medical management, or the short duration of observation during hospitalization.

Studies have also highlighted multiple disparities acting as barriers to adequate pain management in SCD patients, with the most common being racial, social, and geographic disparities and their association with mortality and health outcomes. A 2021 study by Mucalo et al. revealed no significant increase in opioid-related mortality rates in African American individuals with SCD from 2013 to 2019 compared to an increase in opioid-related mortality in African American and Caucasian individuals without SCD [33]. Hood et al. explored the correlation between health-related stigma, racial bias, and health outcomes, discovering that younger African American children perceived more racial bias, which correlated with poorer health outcomes [34]. Other researchers have also documented negative health outcomes associated with race, including subjective discrimination [35, 36]. Additionally, access to urban hospitals has been linked to better outcomes during pain crises, highlighting the importance of healthcare accessibility. Furthermore, a higher level of disease awareness is associated with improved outcomes [37].

Our study addresses long-standing provider concerns and biases regarding poor outcomes and complications associated with opioid use in SCD patients, particularly during VOCs. Patients with SCD are experts on their own disease. NIH guidelines recommend a multidisciplinary approach to SCD pain management involving hematologists, pain specialists, and other healthcare professionals to tailor opioid prescriptions to individual patient needs. This approach not only aims to improve quality of life but also to reduce readmissions and prevent illicit drug use. These guidelines specifically state that providers should determine the analgesic selection based on pain assessment, associated signs and symptoms, outpatient analgesic use, and past experiences with side effects [38]. Further, multiple guidelines from the American Society of Hematology (ASH) also recommend an individualized approach for adequate pain management involving patients in their care [39]. Engaging with patients and prescribing the opioids and dosages that were last effective can help improve patient care outcomes. Extensive education to minimize provider bias in treating SCD patients with high-dose opioids should be implemented irrespective of gender, race, or socioeconomic status. As previously discussed, inappropriate dosages can lead to increased readmissions and possible illicit drug use. Additionally, a multidisciplinary approach and longitudinal follow-up after discharge are essential to ensure adequate peri-discharge outcomes. This approach includes pain specialists, hematologists, and other healthcare professionals working together to manage pain effectively and reduce the risk of complications.

### Limitations

This study has several limitations. Firstly, the reliance on ICD-10 coding may introduce coding errors or misclassifications, potentially affecting the accuracy of our findings. Additionally, we lack data on laboratory results and clinical severity, which could provide a more comprehensive understanding of patient conditions and outcomes. The absence of information on naloxone use is another limitation, as naloxone administration can significantly impact the outcomes of opioid overdose. Furthermore, our analysis does not include long-term follow-up data or readmission rates, limiting our ability to assess the enduring effects of hospitalization and treatment. Although data on socioeconomic status and comorbidities are provided, we lack information on patients’ access to healthcare, which could influence their overall health outcomes. Another limitation is that our database could not distinguish between prescription and illicit opioid sources, which might affect complication rates and outcomes. Other potential confounders, such as variations in hospital practices and regional differences in healthcare delivery, were not accounted for, which could affect the generalizability of our results. Lastly, the study’s retrospective design using administrative data from the NIS limits the ability to establish causality between variables.

### Implications

The findings of our study have several significant implications for clinical practice and healthcare policy. The absence of a significant difference in in-hospital mortality between SCD and non-SCD patients admitted for opioid overdoses suggests that existing treatment protocols are similarly effective for both groups. This challenges any assumptions that SCD patients might inherently have poorer outcomes and underscores the importance of equitable treatment strategies across patient populations. Education of healthcare providers is crucial in minimizing provider bias and ensuring that SCD patients receive care based on clinical need rather than preconceived notions. Addressing potential biases through training and awareness programs can promote more objective and effective treatment decisions. This is particularly important in the management of opioid overdoses, where timely and unbiased interventions can significantly impact patient outcomes. The study also highlights several areas requiring further research, particularly the need for long-term follow-up and data on readmission rates to fully understand the extended impact of opioid overdose treatment in SCD patients.

Recent studies have explored the potential use of opioid antagonists in patients with chronic pain and SCD. These antagonists offer promise in reducing symptoms and improving pain control. For instance, naloxone may enhance pain relief by blocking the inhibition of enkephalin release. Additionally, research by Crain and Shen has highlighted the dual effect of opioids on sensory neurons, suggesting that small doses of opioid antagonists can mitigate opioid side effects and improve pain control by targeting receptors responsible for hyperalgesia [40–43]. Preliminary findings from a pilot study by Koch et al. suggest that simultaneous co-infusion of naloxone and morphine is feasible and does not compromise the analgesic efficacy of morphine while exhibiting no adverse effects [44]. Further investigation is warranted to validate these findings and ascertain the potential of opioid antagonists in enhancing pain management while mitigating concerns regarding opioid overdose.

## Conclusions

We found no significant difference in mortality between SCD and non-SCD patients admitted for opioid overdoses. However, disparities in pain management persist due to biases and stigmatization, underscoring the need for education and awareness among healthcare providers. These findings emphasize the importance of a nuanced approach to managing SCD patients during opioid overdose hospitalizations, focusing on mitigating complications, addressing prolonged hospital stays and higher costs, and reducing healthcare disparities through tailored strategies informed by the unique needs of this population. A multidisciplinary approach to pain management, tailored to individual patient needs and aligned with established guidelines, is crucial for improving outcomes and reducing complications in SCD patients. Addressing these biases and barriers in pain management is essential for enhancing patient outcomes and quality of life. Additionally, further investigation into the use of naloxone alongside opioids in SCD patients is warranted to potentially mitigate pain and reduce overdose rates, thereby improving the quality of life for individuals living with SCD.

## Electronic supplementary material

Below is the link to the electronic supplementary material.

Supplementary Material 1

Supplementary Material 2

## Data Availability

No datasets were generated or analysed during the current study.
