# Supplementary material for: Sickle cell disease and opioid overdose outcomes in the United States: a nationwide analysis
Source: Ann Hematol. 2025 Mar 10;104(3):1551–61. doi: 10.1007/s00277-025-06236-x (PMC12031745; doi:10.1007/s00277-025-06236-x)
Supplement: Supplementary file 1 — Supplementary Material 1 [file 277_2025_6236_MOESM1_ESM.docx]

| **Disease/Procedure** | **ICD-10 Codes** |
| --- | --- |
| Mechanical ventilation invasive | 0BH17EZ,0BH18EZ,5A1935Z,5A0945Z,5A0955Z,5A1945Z,5A1955Z |
| Smoking | F17,F172,F1720,F17200,F17201,F17203,F17208, |
|  | F17209,F1721,F17210,F17211,F17213,F17218, |
|  | F17219,F1722,F17220,F17221,F17223,F17228, |
|  | F17229,F1729,F17290,F17291,F17293,F17298,F17299,Z87891 |
| Vasopressor use | 3E030XZ,3E033XZ,3E040XZ,3E043XZ,3E050XZ,3E053XZ,3E060XZ,3E063XZ |
| Non-invasive mechanical ventilation | 5A09457,5A09458,5A09358,5A09557,5A09558,5A09357,5A0935Z,5A0945Z,5A0955Z |
| Hemodialysis | 5A1D70Z,5A1D90Z,5A1D80Z,5A1D00Z,5A1D60Z |
| Anoxic brain damage | G931 |
| Acute liver failure | K7200,K7201,K712 |
| VTE | I82210,I82220,I82290,I82401,I82402,I82403,I82409,I82411, |
|  | I82412,I82413,I82419,I82421,I82422,I82423,I82429,I82431,I82432, |
|  | I82433,I82439,I82441,I82442,I82443,I82449,I82451,I82452, |
|  | I82453,I82459,I82461,I82462,I82463,I82469,I82491,I82492,I82493, |
|  | I82499,I824Y1,I824Y2,I824Y3,I824Y9,I824Z1,I824Z2,I824Z3,I824Z9, |
|  | I82601,I82602,I82603,I82609,I82611,I82612,I82613,I82619,I82621, |
|  | I82622,I82623,I82629,I82890,I8290,I82A11,I82A12,I82A13,I82A19, |
|  | I82B11,I82B12,I82B13,I82B19,I82C11,I82C12,I82C13,I82C19,I1260, |
|  | I2601,I2602,I2690,I2692,I2693,I2694,I2699,I2609 |
| Chronic pulmonary disease | Elixhauser comorbidities index |
| Diabetes (2 types) | Elixhauser comorbidities index |
| AIDS | Elixhauser comorbidities index |
| Cancer (5 types) | Elixhauser comorbidities index |
| Dementia | Elixhauser comorbidities index |
| Autoimmune | Elixhauser comorbidities index |
| Depression | Elixhauser comorbidities index |
| Hypothyroidism | Elixhauser comorbidities index |
| Obesity | Elixhauser comorbidities index |
| HTN | Elixhauser comorbidities index |
| Alcohol | Elixhauser comorbidities index |
| Cannabis use | F1223,F1228,F1290,F12920,F12921,F12922,F12929, |
|  | F1293,F12950,F12951,F12959,F12980,F12988,F1299, |
|  | F1210,F12120,F12121,F12122,F12129,F12150,F12151, |
|  | F12159,F12180,F12188,F1219,F1220,F12220,F12221, |
|  | F12222,F12229,F12250,F12251,F12259,F12280,F12288, |
|  | F1229,T407X1A |
| Homeless | Z590 |
| AKI | N170,N171,N172,N178,N179,N990 |
| Sudden cardiac arrest | I462,I468,I469 |
| Cardiac dysrhythmia | I470,I471,I472,I479,I480,I481,I4811,I4819, |
|  | I482,I4820,I4821,I483,I484,I4891,I4892,I491, |
|  | I492,I493,I4940,I4949,I495,I498,I499 |
| Opioid poisoning |  |
| Intentional | T400X2A,T400X2D,T400X2S, |
|  | T401X2A,T401X2D,T401X2S, |
|  | T402X2A,T402X2D,T402X2S, |
|  | T403X2A,T403X2D,T403X2S, |
|  | T404X2A,T404X2D,T404X2S, |
|  | T40602A,T40602D,T40602S, |
|  | T40692A,T40692D,T40692S |
| Unintentional | T400X1A,T400X1D,T400X1S, |
|  | T401X1A,T401X1D,T401X1S, |
|  | T402X1A,T402X1D,T402X1S, |
|  | T403X1A,T403X1D,T403X1S, |
|  | T404X1A,T404X1D,T404X1S, |
|  | T40601A,T40601D,T40601S, |
|  | T40691A,T40691D,T40691S |
| Unspecified | T400X3A,T400X3D,T400X3S, |
|  | T401X3A,T401X3D,T401X3S, |
|  | T402X3A,T402X3D,T402X3S, |
|  | T403X3A,T403X3D,T403X3S, |
|  | T404X3A,T404X3D,T404X3S, |
|  | T40603A,T40603D,T40603S, |
|  | T40693A,T40693D,T40693S, |
|  | T400X4A,T400X4D,T400X4S, |
|  | T401X4A,T401X4D,T401X4S, |
|  | T402X4A,T402X4D,T402X4S, |
|  | T403X4A,T403X4D,T403X4S, |
|  | T404X4A,T404X4D,T404X4S, |
|  | T40604A,T40604D,T40604S, |
|  | T40694A,T40694D,T40694S |
| Sickle cell disease | D5700,D5701,D5702,D5703,D5709, |
|  | D571,D5720,D57211,D57212,D57213, |
|  | D57218,D57219,D5740,D57411, |
|  | D57412,D57413,D57418,D57419,D5742, |
|  | D57431,D57432,D57433,D57438,D57439, |
|  | D5744,D57451,D57452,D57453,D57458, |
|  | D57459,D5780,D57811,D57812,D57813, |
|  | D57818,D57819 |
