# Supplementary material for: Sickle cell disease and opioid overdose outcomes in the United States: a nationwide analysis
Source: Ann Hematol. 2025 Mar 10;104(3):1551–61. doi: 10.1007/s00277-025-06236-x (PMC12031745; doi:10.1007/s00277-025-06236-x)
Supplement: Supplementary file 2 — Supplementary Material 2 [file 277_2025_6236_MOESM2_ESM.docx]

| **Supplemental Table 2: Propensity 1:1 Matched Patient Characteristics of Opioid Overdose Patients with Sickle cell disease (SCD+) and Without (w/o) SCD** | | | |
| --- | --- | --- | --- |
| **Characteristics** | **Opioid overdose w/o SCD** | **Opioid overdose and SCD+** | **P-value** |
| n = 2,630 | n = 1,315 (50.0%) | n = 1,315 (50.0%) | -- |
| **Gender (%)** | **n (%)** | **n (%)** | 0.379 |
| Female | 765 (58.17%) | 715 (54.37%) |  |
| Male | 550 (41.83%) | 600 (45.63%) |  |
| **Mean Age Years (SD)** | **Mean (SD)** | **Mean (SD)** |  |
| Female | 42.01 (15.06) | 42.35 (14.29) |  |
| Male | 39.64 (13.58) | 39.39 (13.42) |  |
| **Age Groups (%)** | **n (%)** | **n (%)** | 0.634 |
| 16-29 | 310 (23.57%) | 305 (23.19%) |  |
| 30-49 | 645 (49.05%) | 640 (48.67%) |  |
| 50-69 | 300 (22.81%) | 335 (25.48%) |  |
| >=70 | 60 (4.56%) | 35 (2.66%) |  |
| **Race (%)** | **n (%)** | **n (%)** | 0.682 |
| Asian or Pacific Islander | X* | X* |  |
| Black | 1,155 (87.83) | 1,185 (90.11%) |  |
| Hispanic | 65 (4.94%) | 60 (4.56%) |  |
| Native American | X* | X* |  |
| Other | 55 (4.18%) | 35 (2.66%) |  |
| White | 40 (3.04%) | 30 (2.28%) |  |
| **Median Household Income (%)** | **n (%)** | **n (%)** | 0.450 |
| <=51,999 | 710 (53.99%) | 745 (56.65%) |  |
| 52K-65,999 | 300 (22.81%) | 275 (20.91%) |  |
| 66K-87,999 | 150 (11.41%) | 185 (14.07%) |  |
| >=88k | 155 (11.79%) | 110 (8.37%) |  |
| **Insurance Status (%)** | **n (%)** | **n (%)** | 0.605 |
| Medicaid | 390 (29.66%) | 470 (35.74%) |  |
| Medicare | 705 (53.61%) | 640 (48.67%) |  |
| Other | 15 (1.14%) | 20 (1.52%) |  |
| Private Insurance | 170 (12.93%) | 145 (11.03%) |  |
| Self-pay | 35 (2.66%) | 40 (3.04%) |  |
| **Hospital Division (%)** | **n (%)** | **n (%)** | 0.451 |
| East North Central | 240 (18.25%) | 205 (15.59%) |  |
| East South Central | 90 (6.84%) | 80 (6.08%) |  |
| Middle Atlantic | 165 (12.55%) | 210 (15.97%) |  |
| Mountain | 55 (4.18%) | 45 (3.42%) |  |
| New England | 30 (2.28%) | 25 (1.9%) |  |
| Pacific | 100 (7.6%) | 100 (7.6%) |  |
| South Atlantic | 460 (34.98%) | 445 (33.84%) |  |
| West North Central | 30 (2.28%) | 85 (6.46%) |  |
| West South Central | 145 (11.03%) | 120 (9.13%) |  |
| **Hospital Bedsize (%)** | **n (%)** | **n (%)** | 0.580 |
| Large | 730 (55.51%) | 725 (55.13%) |  |
| Medium | 395 (30.04%) | 360 (27.38%) |  |
| Small | 190 (14.45%) | 230 (17.49%) |  |
| **Hospital Teaching Status (%)** | **n (%)** | **n (%)** | 0.785 |
| Rural | 25 (1.9%) | 35 (2.66%) |  |
| Urban nonteaching | 160 (12.17%) | 145 (11.03%) |  |
| Urban teaching | 1,130 (85.93%) | 1,135 (86.31%) |  |
| **Comorbidities (%)** | **n (%)** | **n (%)** |  |
| HTN | 580 (44.11%) | 555 (42.21%) | 0.660 |
| Diabetes (2) | 165 (12.55%) | 185 (14.07%) | 0.608 |
| Cancer (5) | 40 (3.04%) | 35 (2.66%) | 0.793 |
| Obesity | 195 (14.83%) | 180 (13.69%) | 0.708 |
| Cannabis Use | 135 (10.27%) | 120 (9.13%) | 0.658 |
| Smoking | 505 (38.4%) | 550 (41.83%) | 0.423 |
| Alcohol | 25 (1.9%) | 35 (2.66%) | 0.559 |
| Chronic Pulmonary Disease | 360 (27.38%) | 340 (25.86%) | 0.693 |
| Hypothyroidism | 75 (5.7%) | 70 (5.32%) | 0.848 |
| Autoimmune | 45 (3.42%) | 50 (3.8%) | 0.815 |
| Depression | 335 (25.48%) | 300 (22.81%) | 0.476 |
| AIDS | 15 (1.14%) | 25 (1.9%) | 0.476 |
| Dementia | 25 (1.9%) | 25 (1.9%) | 1.000 |
| Homeless | 25 (1.9%) | 20 (1.52%) | 0.737 |

* Adjusted for Age, Hospital bed-size, Race, Gender, Hospital location, Hospital teaching status, Hospital region, Median household income, Expected primary payer (insurance status), Elixhauser comorbidities.

*****AIDS: Acute immunodeficiency syndrome; HTN: Hypertension; SCD: Sickle cell disease.

* X: Too small to report per NIS data.
